# An empirical study of the impact of generic drug competition on drug market prices in China

**DOI:** 10.3389/fpubh.2023.1146531

**Published:** 2023-05-25

**Authors:** Chen Yina, Liu Pengcheng, Nie Haomiao, Cao Yang

**Affiliations:** School of International Pharmaceutical Business, China Pharmaceutical University, Nanjing, China

**Keywords:** generic drug, competition, drug price, China, pharmaceutical industry

## Abstract

**Introduction:**

Generic substitution is encouraged to reduce pharmaceutical spending in China, and with incentive policies, the market size of the generic drug continues to rise. To find out how the generic competition affects drug price in this area, this study examines how the quantity of generic drug manufacturers can influence average drug price in the Chinese market.

**Methods:**

This study uses a rigorous selection of drugs from the 2021 China’s National Reimbursement Drug List (NRDL), and uses drug-level fixed effects regressions to estimate the relationship between competition and price within each drug.

**Results:**

We note that drug prices decline with increasing competition in the Chinese market, but not in a perfectly linear manner, with marginal price declines decreasing after the fourth entrant and “rebounding” at subsequent entrants, especially the sixth.

**Discussion:**

The findings suggest the importance of maintaining effective competition between suppliers to control prices, and that the government needs to further control generic pricing, especially for late entry generics, to ensure effective competition in the Chinese market.

## 1. Introduction

Generic substitution is among the best policy options used by health authorities in many countries to maintain drug prices reasonable level and control health care costs (1, 2). To promote the substitution for generic medicines, Chinese government has focused on reducing health expenditures and improving access to medicines by introducing a series of favorable policies in recent years to encourage the development and use of high-quality generic drugs, such as abolishing government pricing of drugs (3), reforming generic drug standards (4), and comprehensively promoting the consistency evaluation of generic drugs (5), to ascertain the quality and efficacy of generic drugs (6). At the same time, patents for the world’s top-selling medicines are concentrated at expiry (7). Increased generic competition has created opportunities for low-cost generic drugs to enter and scale up their use (8). As a result of policies and the successive expiration of patents for originator drugs, the size of Chinese generic market continues to increase. These low-cost generic drugs are expected to further moderate the growth of China’s basic medical insurance fund expenditures. China’s market share and influence in the world are continuing to rise, and it has now become the second largest pharmaceutical market in the world after the United States, and Chinese drug prices are receiving widespread attention from the global market.

However, we do have some concerns about competition and price in the generic drug industry in China. In the Chinese market, although generic companies have large production capacity, lower costs for raw materials and labor, and a huge amount and market for generic drugs, etc. (9). Unfortunately, the quality of generic drugs is not high, and we doubt that generic drugs in the Chinese market will be able to replace the original drugs in the real world (10, 11), or if patients have an inflexible demand for brand-name drugs, which may lead to higher prices for the same after patent expiration, offsetting loss of profit margin from the market (12). On the other hand, although there are 2,210 ANDA applications for generic drug registration in China in 2021, representing a 81.30% increase over the previous year (13). we find that currently approved generic drug submissions are very homogeneous (14), mainly for existing generic drugs, and we doubt the newcomers will be able to reduce drug prices as much as the former (15). Therefore, further research is required on the relationship between the number of competitors and prices in China.

Considerable literature exists with differing estimated effects on the relationship between the number of competitors and prices in generic drug markets. Frank and Salkever (1995) (16) examined the effect of the number of generic sellers (N) on prices using IMS data from 1980 to 1991 for a sample of 32 drugs that lost patent protection during 1984–87. The results indicate that each entrant would reduce the price of generic products by 5.6–7.2%. Berndt, Conti, and Murphy (2017) (17) use IMS national sales perspective data from the fourth quarter of 2004 to the third quarter of 2016 and find that the elasticity of price with respect to the number of manufacturers ranges from −0.710 to 0.777, assuming an estimated elasticity of 0.75, an increase from two to three manufacturers implies a 30% price decrease, while when molecular fixed effects are included, the study shows that an increase from two to three manufacturers implies a price decrease of approximately 15%. Nguyen (2021) (18)used Medicare Part D drug events (PDEs) from 2007 to 2018, using drug-level fixed and random effects to estimate the relationship between competitors and prices within each drug, the study found that drug prices decreased as the number of competitors increased, with prices decreasing by 20% in markets with three competitors; in markets with 10 or more competitors, prices continued to decrease by 80% relative to pre-generic entry prices. Kesselheim (2017) (19) examined the relationship between the number of generic manufacturers and the relative prices of generic versus brand-name drugs using 2008 MarketScan commercial claims data, showing that the relative price of drugs with one generic competitor declined to 87% and then to 77%, and further to 60% with three manufacturers; the study also found that generic entry had the most significant impact on the top three manufacturers, with marginal relative prices decreasing as the number of manufacturers increased. Reiffen and Ward (2005) (20), using IMS data on 31 drugs that were off-patent in the late 1980s and early 1990s, with competition represented by a dummy variable for generic sellers, found a negative relationship between price and the number of firms, and that as the number of firms increased, an additional firm’s marginal effect tends to decrease. In the study on the Chinese market, Dave, Hartzema and Mingyue Zhao and Jing Wu (2016) (21) used data from the Urban Employees’ Basic Medical Insurance (UEBMI) database in Tianjin, and Jing Wu et al. (2014) (22) used data from 2002–2005 from 11 public tertiary hospitals in three Chinese cities, studied the role of regulation and competition in the drug market, and they both found that generic competition still lowers prices under government regulatory policies in China.

The general conclusion of these studies is that generic competition is closely related to drug prices, but there are some differences in the level of specific correlation, and the differences in these outcomes may be attributed to the different drug samples studied, the various periods, the different national situations, and the selection of statistical models. Few such studies exist on the relationship between generic competition and drug prices in the Chinese market, and the existing literature relates primarily to the relationship between price and competition under the government’s price regulation policy in China, which may not reflect the actual situation of the Chinese market at this point. This study will fill the current lack of research and enrich the pricing research system in China, which may have a greater reference value in formulating drug policy in China, especially drug pricing policy. The findings of this study are relevant for other countries since concerns about the effectiveness of generic substitution and harmonization of generic drug applications are not unique to China. These two problems exist in most countries around the world, especially in developing countries with similar conditions to China (15, 23).

In this study, we investigate the relationship between competitors and prices within each drug using data for 101 drugs that lost their patents in the 2021 China’s NRDL, using a linear regression model with drug-level fixed effects. We examine the relationship between competition and generic prices using two measures of competitive effectiveness: (1) the number of generic drug manufacturers as a continuous variable, and (2) a dummy variable representing the number of generic competitors. We want to address two key issues identified above. First, does the average market price of a drug vary based on number of generics on the Chinese market? Second, does the average market price of a drug continue to decrease with each new market entrant after the original drug has lost its patent exclusivity, or does it stabilize after the early participants?

## 2. Data and methods

### 2.1. Data

This study refers to the original drug for the period of patent protection for invention granted by the Intellectual Property Office of the People’s Republic of China or the drug labeled as originally developed in the National Development and Reform Commission documents. Generic drugs are drugs containing the same active ingredients as the product being copied, and with the same indications, dosage forms, specifications, routes of administration, bioequivalence and quality in line with the same requirements as the product being copied.

Since China’s NRDL is unified and adjusted by the National Healthcare Security Administration, drugs that meet the basic conditions of clinical necessity, safety and effectiveness are included in the NRDL for management, and the included drugs is in a high number, covering a wide range of therapeutic areas, and they are all common clinical supplies. It is a good representation of the actual situation of China’s drug market. To investigate the relationship between the number of drug manufacturers and prices in the mainland China market, we screened drugs from the 2,860 drugs (1,486 western drugs and 1,374 proprietary Chinese drugs) in the 2021 China’s NRDL. Our exclusion criteria were as follows.

1. Exclude competition in drugs before 2015, i.e., exclude generic drugs that first entered the market before 2015, because government pricing of drugs was abolished in mainland China only after 2015 (3).
2. Exclude biological drugs. Because of the complex macromolecular structures and manufacturing processes of biologics, biosimilars cannot be exact replicas of the originator drugs as chemical generics, and usually require animal testing and clinical studies to confirm similar efficacy before they can be marketed. Moreover, due to high research and development costs, stringent manufacturing conditions, high marginal manufacturing costs, and blurred patent boundaries, biosimilars have higher competitive barriers, so the market pricing logic of biosimilars is usually different from that of chemical generics (24), and the price elasticity of biosimilars is smaller compared to chemical generics.
3. Exclude drugs less than four quarters after the first generic entered the market to observe the relationship between the number of drug manufacturers and prices over time.

**Figure 1:**
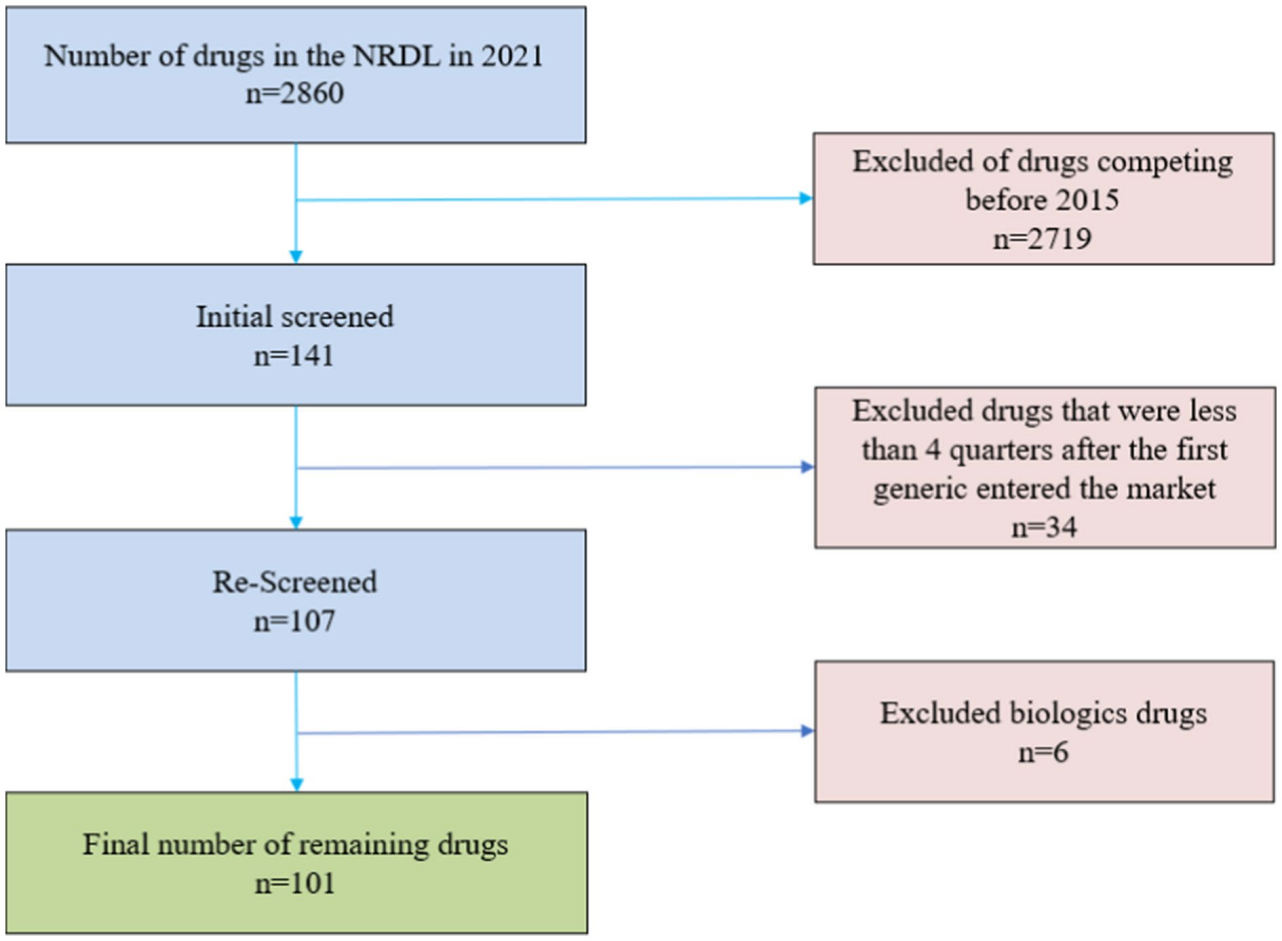
Screened flow chart.

All drugs included in the study after passing the exclusion. The drugs in our study were defined by a combination of active ingredient, route of administration and dosage form. We ended up with a total of 101 drugs screened, the specific screening process is shown in Figure 1. The unit of analysis was a drug-quarter (active ingredient-dosage form-quarter), an approach that significantly reduces the number of observations compared to implicitly treating each month as an independent observation, which tends to reduce the statistical significance of our results, but the final results show that we were still able to find significant competitive effects.

The baseline price for each drug is the average price for the four quarters before a generic entered the market for that drug to ensure exogeneity of the baseline price. This baseline price is the baseline against which we compare the trajectory of price changes across quarters since the first generic entered. As China fully implements generic drug consistency evaluation after 2016 (5), at the drug level, we consider the quality and efficacy of both originator and generic drugs to be consistent, and therefore we use the average market price that includes both originator and generic drugs as the independent variable in our study, rather than either one alone.

In China’s drug market, there are many different types of prices, such as factory prices of enterprises, hospital purchase prices, national volume-based procurement prices, medical insurance negotiation prices, retail prices of retail pharmacies, etc. However, for now, many of these prices are already equivalent. In 2017, China required all public hospitals to abolish drug markups and not to sell drugs at a markup on top of factory prices (25), and in most cases, hospital procurement prices are equivalent to corporate ex-factory prices/collection price/negotiated price, so we believe that using public hospital procurement prices is more reflective of the real competition in the drug market than retail pharmacies.

The drug price datum are the average quarterly prices of all manufacturers’ tenders in each Chinese province from the first quarter of 2015 to the second quarter of 2022 under each generic drug obtained from the Pharnexcloud website. Pharnexcloud is a one-stop search platform for China’s domestic pharmaceutical industry, in which the bidding database integrates and includes the drug procurement prices of most public hospitals in 31 provinces and municipalities in China. All of its datum come from the official government websites of each province, and the website is only responsible for integration and inclusion. For cross-year comparisons, all prices use an inflation rate of 2.1% (26) (expressed as the average of the Consumer Price Index for the last 3 years published by the National Bureau of Statistics of China) discounted and adjusted to the prices in the 2nd quarter of 2022.

We prefer to use the number of producers to indicate generic competition as opposed to the drug’s labeler, because even the original drug producer may commission other firms to bid, which usually does not generate price competition. This means that using the labeler causes competition to emerge earlier and has a smaller estimated effect. We consider a drug manufacturer as entering if it has positive sales in the first month after at least one quarter of zero sales, and the dependent variable of the study is the number of generic drug manufacturers entering the Chinese market for that drug from 2015–2022.

### 2.2. Methods

#### 2.2.1. Variables

The main dependent variable in this study is the ratio of the average market price after market entry with a generic drug relative to the baseline price of the originator before generic entry. The main independent variable is the number of manufacturers of the generic drug entering the Chinese market.

We use both the number of producing manufacturers as a continuous variable and a series of dummy variables to account for competition. This is because when the number of producing manufacturers is used as a continuous variable as an explanatory variable, the implicit assumption is that the effect of each increase in the number of manufacturers is the same regardless of the initial number of manufacturers (20). Therefore, we introduce a series of dummy variables to represent the number of manufacturers in the China market to examine how the marginal effect of an additional number of drug manufacturers on the average market price varies with the number of generic firms.

We used fixed effects and random effects applied at the drug level to control for different drug factors. Using the Hausman test, we finally chose fixed effects to control for the potential endogeneity of the relationship between the number of competitors and price (27).

In addition, since there are some literatures indicating that competition is intense for a period of time after the expiration of a drug patent and later diminishes over time (19, 20), we follow each drug for at least 4 quarters after the first generic entry to see if there is an impact on drug prices over time in the Chinese drug market and if the relationship between the number of generic manufacturers and prices changes over time as in other countries.

Also, since other factors besides the number of generic manufacturers may affect the price, we also tried to introduce some control variables to our model.

1. Market size (16, 18, 19) expressed as the number of units sold for the generic drug (both originator and generic) in 2022, in million units. We believe that if the market size is small, it may enter saturation competition soon, and a larger market size may be in a competitive market for a long time; however, at the same time, a larger market will attract more suppliers to enter, while a smaller market may not be as attractive.
2. National negotiation of drugs (28), a policy variable, is represented as a dummy variable. Since 2016, China has adopted a drug national negotiation policy in order to reduce drug prices, and the drugs are covered by China’s NRDL after successful negotiations, which usually result in significant reductions in drug prices.
3. Volume-based procurement of drugs (29), a policy variable, expressed as a dummy variable. With the government as the largest buyer, consolidating the number of drugs used in public hospitals in order to obtain drug discounts and reduce drug prices, the policy of centralized quantity purchasing of drugs usually results in a significant reduction in the average market price of drugs. Due to the great number and complexity of provincial alliances for volume-based procurement at the local level, this study refers only to national-level volume-based procurement of drugs.

We believe that both drug national negotiation and volume-based procurement are only transient policy effects, although they can significantly reduce prices in the current period, they do not exert sustained downward policy pressure on drug prices (basically do not affect the trend of price changes) (30, 31). After the point in time of drug national negotiation and volume-based procurement, competition is still the main factor that makes prices lower. Controlling for national negotiations and volume-based procurement will allow identification of the *ceteris paribus* effects of the number of generic manufacturers.

#### 2.2.2. Models

We use two methods to describe the number of competitors (n), the independent variable in this study: (1) using the number of all generic drug manufacturers in the market n; (2) using a series of dummy variables N_j_ to describe the number of competitors.

Our first approach is used to explore whether the number of competitors n has a significant effect on relative prices, controlling for the relevant variables. To address this question, we develop a linear regression model based on the international literature on the subject and taking into account the unique national context of China.


$$\frac{P_{ift}}{P_{ifu}}=\alpha_{0}+\alpha_{1}\left(n_{ift}\right)+\alpha_{2}time+\alpha_{3}Size+\alpha_{4}{\mathrm{Ne}}_{ift}+\alpha_{5}VBP_{ift}+\theta_{if}$$

where:

i denotes the drug (active molecule), f denotes the dosage form, t denotes the number of quarters since the drug became available as a generic, and u is the number of quarters before the availability of a generic.

P_ifu_ is the average price of originator drug with active molecule i in dosage form f in the u quarters before a generic becomes available.

P_ift_ is the average market price of a drug with active molecule i in dosage form f in quarter t.

P_ift_/P_ifu_ is the dependent variable in this study, which is the ratio of the average market price of pharmaceuticals after market entry relative to the baseline price of originator drugs before generic entry. n_ift_ is the number of generic manufacturers of drugs with active molecule i in dosage form f in all markets in quarter t and is the main independent variable in this study.

time is the number of quarters after the entry of the first generic version of the drug with active molecule i in dosage form f to examine whether there is an effect on drug prices over time.

Size is the number of drugs with active molecule i in dosage form f sold in quarter t to control the market size.

Ne_ift_ indicates whether a drug with active molecule i dosage form f performs national negotiations in quarter t, expressed as a dummy variable.

VBP_ift_ indicates whether a drug with active molecule i in dosage form f performs centralized volume purchasing in quarter t, expressed as a dummy variable.

θ_if_ is a fixed effect at the drug dosage form level, which includes all variables that do not vary over time to control for exogeneity of the model.

α is the coefficient of the respective variable under the first method, by which we can know whether the baseline price ratio of the dependent variable increases or decreases when the respective variable changes, and how much is the magnitude of the change.

Our second approach is used to investigate how the marginal effect of an additional competitor on the price of a drug varies with the number of generic firms. The model we develop is as follows.


$$\frac{P_{ift}}{P_{ifu}}=\beta_{0}+\overset{n}{\underset{j=1}{\sum }}\gamma_{j}N_{j}+\beta_{2}time+\beta_{3}Size+\beta_{4}{\mathrm{Ne}}_{ift}+\beta_{5}VBP_{ift}+\theta_{if}$$

β is the coefficient of the respective variable under the second method. All control variables are the same as in method one except for the representation of the independent variable generic drug manufacturer.

N_j_ is a dummy variable equal to 1 when there are j generic manufacturers and 0 otherwise. By setting dummy variables, we are able to obtain coefficients for all generic drug manufacturers N_j_ within the range of values of j in order to study the effect of an additional drug manufacturer on the average market price.

## 3. Results

### 3.1. Descriptive statistics

After a rigorous selection of nadir criteria, our analysis ultimately includes 101 drugs with 1,595 observations with at least one generic vendor competitor over the 2015–2022 q2 period. There were 2 traditional Chinese drugs and 99 western drugs in our study. Our study drugs include 12 negotiation drugs, 32 centralized procured drugs, and 7 that have both participated in national negotiations and in national volume-based procurement.

**Table 1:** Number of largest generic manufacturers of drugs by market size.

	Small (<10)	Medium (10–100)	Large (>100)	Total
1	9	2	3	14
2	2	7	6	15
3	4	5	4	13
4	1	6	2	9
5	3	4	7	14
6	2	4	5	11
7	4	1	2	7
8	1	0	1	2
9	1	3	3	7
10 (and above)	2	2	5	9
Total number of drugs	29	34	38	101
Average number of generic drug manufacturers	4.17	4.50	5.24	4.68

China State Drug Administration, Pharnexcloud. Based on the identified study drug (at the molecular-dosage form level), the number of generic manufacturers of the drug entering the Chinese market through the second quarter of 2022 was queried in the State Drug Administration of China and the Pharnexcloud drug price database, and the market size of the drug was queried in the Pharnexcloud public hospital chemical database in key cities.

We expect the number of generic vendors to be highly correlated with the size of the market in which they compete, i.e., larger markets will attract more vendors (32), we divide the market into small, medium, and large categories, according to the average of the number of generic drugs sold at the time of their first market entry and the last observation due to the long time span, the high variability in market size, and the excessive price differences between drugs.

Table 1 shows the maximum number of competitors for the studied drugs by market size, using the number of largest competitors in the market at the last quarter of the study (2022q2) as a statistical criterion. In our study, the distribution of drugs in small, medium and large markets was relatively uniform, with the majority of drugs in small markets (sales volume less than 100,000) having only one competitor, the majority of drugs in medium markets (sales volume between 100,000 and 1 million) having less than five competitors, and the majority of drugs with more than 10 competitors. Most of the drugs with more than 10 competitors are in large markets (sales volume > 1 million).

**Table 2:** Number of study drugs by dosage form.

Dosage form	Number of study drugs
Tablets	41
Injection	29
Capsules	9
Sustained-release controlled-release dosage forms of drugs	5
Oral liquid formulations	4
inhalants	4
Granules	2
Disintegrating Tablets	2
Eye Drop	1
ointment	1
creams	1
powders	1
pills	1

In addition, we also counted the dosage forms (Table 2) and indications of the studied drugs (Table 3). The drugs in this study were mainly tablets and injectables, accounting for 40.59 and 28.71% of all studied drugs. The indication profiles were expressed as ATC-3 classification (pharmacological classification) (some drugs were only available in the China’s NRDL at ATC-2 therapeutic classification), ATC classification is the official WHO classification system for drugs. Our 101 study drugs covered 52 indications, with the other antineoplastic agents (9.90%) and antithrombotic agents (7.92%) being the most prevalent among all drugs studied, but with a small proportion of single therapeutic areas, which makes our study less prone to indication bias and more representative of the actual situation in the Chinese drug market.

**Table 3:** Number of study drugs counted by ATC-3 class (classes with ≥ 3 drugs).

ATC-3	Indication	Number of drugs
XL01X	Other Antineoplastic Agents	10
XB01A	Antithrombotic Agents	8
XA10B	Blood Glucose Lowering Drugs, Excl. Insulins	6
XN03A	Antiepileptics	5
XN05A	Antipsychotics	5
XL04A	Immunosuppressants	4
XN06A	Antidepressants	4
XN04B	Dopaminergic Agents	3

### 3.2. Competition effectiveness assessment – measured by the number of competitors in Method 1

This section discusses the relationship between the number of generic drug manufacturers and prices, a result obtained using Method 1. The model *p*-value is 0.000 (< 0.05), the within R-sq value is 0.692 and the adjusted R-sq value is 0.671, which indicates a good fit of the model.

Table 4 shows the effect of the number of generic drug manufacturers on drug prices based on Method 1. The results of our study show that lower drug prices are associated with more generic competition and longer competition time. The coefficient of the variable “number of generic manufacturers n_ift_ “indicates that after the first entry of generics, each entry decreases the average market price ratio to the baseline price of the originator before the entry of generics by about 2.50%. The coefficient on the time variable time indicates that the relative price of a generic drug decreases by about 1.02% each quarter after its first entry. The variable “National Negotiations Ne_ift_ “indicates that the implementation of national negotiations leads to a 21.00% reduction in relative prices. The “Centralized Volume Purchasing VBP_ift_ “variable, on the other hand, indicates that the implementation of drug volume purchasing would lead to a 17.96% reduction in the average market price of drugs. All of these price influences are statistically significant. These estimated impacts suggest that in a market with about 10 competitors, the price of a generic drug is expected to be about 25.00% lower than the price of a branded drug before generic entry.

**Table 4:** Effect of the number of generic drug manufacturers on drug prices (based on Method 1).

	Coefficient values	*p*-value
Number of generic drug manufacturers (n_ift_)	−0.0250505	0.000
time	−0.0101961	0.000
National Negotiation (Ne_ift_)	−0.2099628	0.000
Volume-based procurement (VBP_ift_)	−0.1795535	0.000
size	0.00009927	0.001
constant	3.267597	0.000

Regressions were performed using stata 16.0 for all study drugs under drug molecule level fixed effects with the relative price ratio in the drug market as the dependent variable and the number of generic drug manufacturers, etc. as the independent variables. The number of obs is 1,595, the within R-sq value is 0.692 and the adjusted R-sq value is 0.671.

### 3.3. Competition effectiveness assessment – measured by a set of dummy variables based on the number of competitors in Method 2

Method 2 uses a series of dummy variables to represent the number of generic drug manufacturers under each drug, with the number of generic drug manufacturers at 0 as the control group, when there are no generics and only one supplier of the original brand product in the market, so that the coefficients of the dummy variables have the meaning of how much the market price decreases relative to the case when there are no generics. The use of dummy variables helps to reveal how the marginal effect of an additional competitor on the price of a drug varies with the number of generic firms. The model *p*-value is 0.000 (<0.05), the within R-sq value is 0.723 and the adjusted R-sq value is 0.702, which indicates a good fit of the model. Compared with Method 1, the adjusted intergroup R-sq values of Method 2 are higher, which indicates that model 2 is a better fit, meaning that the relationship between the average market price and the number of competing manufacturers in the actual China drug market is closer to nonlinearity.

**Figure 2:**
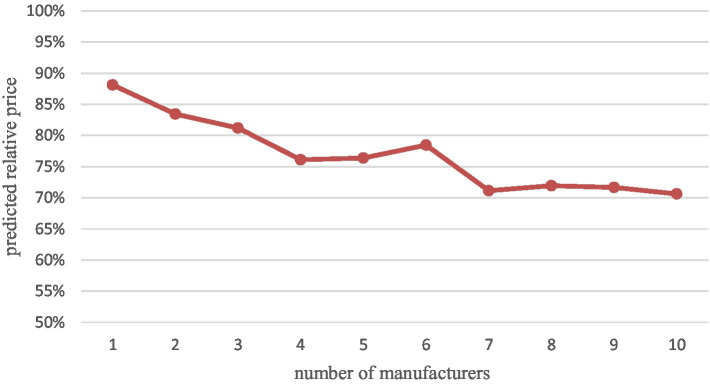
Relative prices predicted according to Method 2.

Table 5 shows the effect of the number of generic drug manufacturers on drug prices based on Method 2. Our results show that the average market price continues to decline as the number of competitors increases. After the entry of the first generic, the relative price decreases by 23.89% in the market with four generic manufacturers, which means that the average market price with four generics is only 76.11% of the price with no generics. Compared to the results of Method 1 (where each competing manufacturer’s entry leads to a 2.50% market price reduction), the relative price reductions for the top 4 generics are very rapid and far exceed the overall magnitude. Meanwhile, after the presence of 4 generic manufacturers, the average market price decline slowed to a lower level than the overall rate. In addition, some drug prices rebounded, i.e., new generic manufacturers entered, but relative prices increased compared to the previous ones, especially when the sixth (albeit insignificant) generic manufacturer entered. The largest relative price decline ratio (29.39%) was observed in the market with 10 generic manufacturers. Through the Figure 2 we can more visually see the relative price decline trend.

**Table 5:** Effect of the number of generic drug manufacturers on drug prices (based on Method 2).

		Coefficient value	*p*-value
Number of generic drug manufacturers (N_j_)	1	−0.118922	0.000
	2	−0.1654992	0.000
	3	−0.1879174	0.000
	4	−0.2388876	0.000
	5	−0.2360711	0.000
	6	−0.2154436	0.000
	7	−0.2885595	0.000
	8	−0.2806422	0.000
	9	−0.2834121	0.000
	10 (and above)	−0.2939239	0.000
Time		−0.0087823	0.000
National Negotiation (Ne_ift_)		−0.1445838	0.000
Volume-based Procurement (VBP_ift_)		−0.1828591	0.000
Size		−0.0000911	0.001
Constant		2.998563	0.000

Using the relative price ratio in the drug market as the dependent variable and dummy variables such as the number of generic manufacturers as independent variables, all study drugs were regressed using stata 16.0 at the drug molecular level fixed effects, with *N* = 0, i.e., when no generics were available and only one supplier of the original brand product was used as the control group. The number of obs is 1,595, the within R-sq value is 0.723 and the adjusted R-sq value is 0.702.

### 3.4. Subgroup analysis of different therapeutic areas

**Figure 3:**
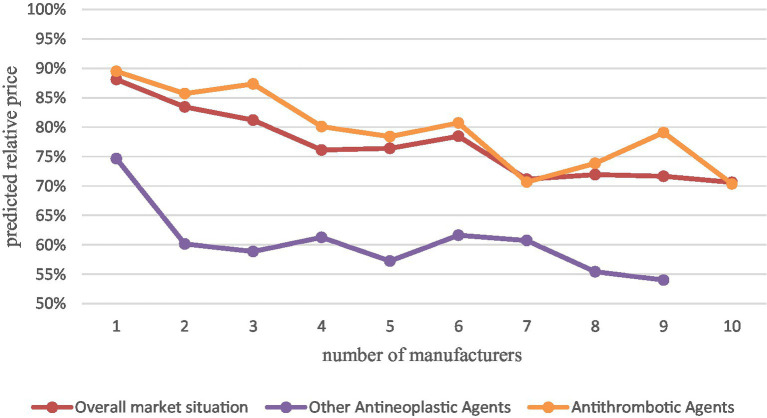
Comparison of other antineoplastic agents and antithrombotic agents with the overall market situation.

Since our data cover a relatively scattered therapeutic area (52 pharmacological classifications) with a small proportion of drugs in a single therapeutic area and most therapeutic areas have only one or two drugs, most of them may not be significant if subgroup analysis of different therapeutic areas is performed, therefore, we performed separate subgroup analysis with two therapeutic areas that have more drugs, the other antineoplastic agents (10, 9.9%) and antithrombotic agents (8, 7.92%) were analyzed in separate subgroups (Figure 3).

The results of the analysis showed that with the increase of generic manufacturers, the average market price of other oncology drugs decreased significantly more than the overall China’s drug market situation, especially after the first two generics entered, with a relative price decrease of 40%. while the average market price of antithrombotic drugs decreased close to the overall market situation and slightly less than the overall market situation. This indicates that for different therapeutic areas, drug price elasticities differ. However, the sample size was insufficient to study it in depth, which may be a direction for subsequent research.

## 4. Discussion

In this study, 101 drugs from the 2021 China’s NRDL were screened by strict nadir criteria, including all their manufacturers who entered the Chinese market from the first quarter of 2015 to the second quarter of 2022, and their post-entry prices were collected to obtain drug price data. The drug price data is the ratio of the average quarterly price to the baseline price of all drug tenders in each Chinese province from the first quarter of 2015 to the second quarter of 2022 under each generic name drug obtained from the Pharnexcloud website. This study applies regressions with drug-level fixed effects using two different methods to construct the model to focus on the effect of the number of competing manufacturers on the price for each drug, while controlling for other factors affecting the model using variables such as time variables, market size, national negotiations, and drug banding.

First of all, We finally found that in the Chinese market, the number of generic manufacturers is always significantly negatively correlated with drug prices, that is, as the number of generic drug manufacturers increases, the average drug market price continues to decrease, which is similar to the situation in other countries in the literature, indicating that even though the quality of generic drugs is not high, most patients’ demand for originator drugs is not very rigid, and most originator drugs do not raise their prices after patent expiration, and the generic substitution strategy in China has had some effect in reducing drug prices and saving health care costs. And the results of both approaches suggest that the erosion of price cost margins caused over time has led to a reduction in prices. In addition, for different therapeutic areas, the impact of generic manufacturers’ entry on the average market price may be different, and for special therapeutic areas such as cancer, the price decrease may be greater than the average market situation, which may be due to the high demand and low price elasticity of cancer patients, so the original cancer drugs are priced high to earn excess profits, and the first few generics enter before the price returns to the drug itself real value. This reinforces the significance of generic substitution.

Second, we also find that there are some differences in the Chinese drug market from other countries. According to our findings, the effect of the number of generic manufacturers in the Chinese market on drug prices is not perfectly linear, but shows a sharp decline followed by a gradual leveling off, and is smaller than the range estimated by most of the literature (see Table 6 for results from other literature). We suspect that the lower patent prices in China compared to the U.S (33) may leave little room for further price reductions for originator drugs after patent expiration, and that even with generic manufacturers, originator drugs do not necessarily lower prices, resulting in smaller average market price declines than in other countries. However, we find that drug price reductions through supply-side market competition are limited, but the coefficients on the two policy variables suggest that price reduction pressure from the buyer’s perspective through health insurance can have a very significant effect, and if health care purchasers were to promote intense competition for price discounts, we would expect a greater decline in average market prices than the existing extent.

**Table 6:** Results of this study and related references.

Number	Author	The impact of generic drugs entering the market	
		3–5	6–10
1	Frank and Salkever (16)	17–35%	56–72%
2	Reiffen and Ward (20)	15% (3)	30–40% (10)
3	Chintan et al. (19)	40% (3)	79% (10)
4	Berndt et al. (17)	15% (3)	/
5	Nguyen et al. (18)	20% (3)	80% (10)
This study	Yina Chen and Pengcheng Liu	18.79–23.89%	21.54–29.39%

And, unlike some studies that suggest that marginal prices continue to decline as the number of competitors increases, our study supports the view of another part of the literature that marginal price declines after about four to six entrants. We find that in markets with about four competitors, relative prices decline by 23.89% (the expected price ratio of the average market price to the average pre-generic entry brand price is 76.11%). In contrast, in markets with 10 competitors, the price decreases by a maximum of 29.39% relative to the pre-generic entry price. This suggests that the entry of the first few generic manufacturers can significantly reduce drug prices, but later generics do not provide the expected price reductions.

Last but not least, we also found that the impact of the number of generic manufacturers on prices in the Chinese drug market is subject to a “rebound” phenomenon, i.e., as the number of generic manufacturers increases, prices fall sharply and then rebound slightly. After analyzing the raw data, we found that this is due to the fact that some generic drug manufacturers’ pricing is much higher than that of the original drug, which increases the average market price. By examining the relevant literature, we found that there was also a small rebound in some international literature (19), However, none of them analyzed the reasons for it. This rebound phenomenon is particularly prominent in the Chinese drug market, and we speculate that there are two possibilities for this situation. First, the rebound phenomenon may be due to the special pattern of the Chinese drug market. In the Chinese market, public medical institutions are the main sales channel for drugs. However, there is a limit to the number of drug specifications in public hospitals, which means some hospitals that are already fully equipped with drugs need to transfer the corresponding number of drugs if they want to add new drugs. And usually, all drugs in public hospitals must go through a complex review process by the hospital’s pharmacy management committee, and the frequency of most tertiary hospitals’ pharmacy committees is once a year or once every 6 months. When several generic drugs enter the market, they may have basically met the market demand of public hospitals, and the market is almost completely taken up, especially for the national negotiation drugs and centralized quantity procurement drugs. As the selected varieties acquired most of the public (and private) markets, the unselected varieties and subsequent generic entrants divide the remaining small market. Competition is more intense at this time, and later generic manufacturers may not get the expected feedback by simply lowering their list prices. If later generic entrants have to offer rebates (aggregate discounts) in order to gain larger business volumes from hospitals (or to even enter the hospital market), they may increase their list prices to the point that generic prices could be two to three times that of originator products. Alternatively, subsequent generic manufacturers may be tempted to promote their products as a differentiated alternative to justify a higher price. However, if health care payers do not consider this differentiation worthwhile, then the price premium is no longer justified. Later entrants will have no choice but to lower their prices to match those of their competitors or lose sales. Subsequently, even if generic manufacturers do not change their pricing strategy, as new generic products entered the market at low prices, they brought the average market price back to normal levels, thus leading to a “rebound” phenomenon in our results. A related study found that in drug markets with fuller generic competition, competition among companies forced illegal rebates to occur and dominate, resulting in smaller absolute price elasticities for generics, which supports our speculation (34).

The second possibility is that the entry of the first few generic drug manufacturers has significantly reduced drug prices, especially under the national drug price negotiation and the volume-based drug procurement policies, the market structure has changed, and health insurance as the largest buyer is committed to suppressing drug prices, and the continuous downward pressure on prices usually drives manufacturers to suppress costs, but the generic drug consistency evaluation policy sets a minimum limit for generic drug quality, in order to keep reaching normal drug quality, making it impossible for companies to ensure profitability and produce in an unstable or unsustainable economic situation, which could lead to supply disruptions and shortages of drugs that could be followed by a retaliatory price rebound. Afterwards, prices returned to the normal range as new generic manufacturers came to market to relieve supply pressure. Frank (2021)‘s study (35) shows some upward trends in generic prices in the U.S. markets that corresponded to periods when the shortages and recalls were at their highest levels. However, we did not find a similar empirical study in China market, which may be a direction for further research to follow.

Our findings also have some policy implications. First, we support the policy of generic drug consistency evaluation and generic substitution, and promote effective market competition plays a similar role as expected in lowering drug prices, reducing pharmaceutical cost expenditures, and saving health insurance funds, and governments can vigorously promote generic substitution while ensuring drug quality. And the price reduction pressure exerted by health insurance as a buyer can bring significant results, and governments must actively promote competition for price discounts while ensuring the quality of drugs. Second, we found that the first few entering generics can significantly reduce drug prices, while later generic entrants do not significantly reduce market price. Governments should pay attention to the review and approval of the first few generics, especially for drugs without competitive manufacturers or with insufficient competition. Furthermore, governments must pay attention to supply-side policies, monitor drug costs, and further regulate and control generic drug pricing, both to prevent late-entry generics from reserving rebate space to set high prices and to prevent drug shortages due to continued downward pressure on prices, and to maintain effective and adequate as well as transparent and fair market competition.

There are still some limitations of our study and further research is needed in the follow-up. First, although we screened among 3,260 drugs in the drug list, we ended up with only 101 representative drugs, which is only a small fraction of China’s large drug market. Subsequent inclusion of data on more drugs and tracking the entry and exit of their generic manufacturers, as well as inclusion of interaction variables between different dosage forms under the same generic name and different active molecules in the same therapeutic area to control for the impact of other types of competition will help us further understand the impact of competition on drugs. Second, although our study chose to use the mean method to measure the average drug market price due to the lack of clarity regarding the market share of each manufacturer under the same generic name in China, this may be very crude, as opposed to using a price index or a price weighted by volume, which may be more appropriate and should be used in subsequent studies if possible. Finally, the lack of data on rebates for drugs leads to our inability to measure the true price of drugs, which implies that the actual effect of generic competition may be larger than the results estimated in this study, especially for generic drugs that enter the market later, the difference in pricing strategies determines that their prices are likely to be high, leading to our underestimation of the effect of competition. However, the potential underestimation of these effects is mitigated by the fact that rebates may also be paid for originator drug pricing, and our findings still have some credibility and reference value.

## 5. Conclusion

In summary, the results of our study provide clear evidence in favor of policies that promote generic entry. However, the findings suggest that marginal prices will continue to fall as the number of generic drug companies entering the market increases, and efforts to maintain effective competition among suppliers are important to control prices. In addition, other controls on generic drug price, especially for late-entry generics, may be necessary to ensure effective competition in the Chinese market.

## Data availability statement

The original contributions presented in the study are included in the article/Supplementary material, further inquiries can be directed to the corresponding author.

## Author contributions

CYi and NH conceived the study based on the input from LP and CYa. CYi was responsible for data analysis, model building, and the main writing of the article. LP provided the research ideas and was responsible for the review of the study. NH was responsible for data collection, organization and analysis, and article writing. CYa provided the research framework and was responsible for the supervision and review of the study. All authors contributed to the article and approved the submitted version.

## Conflict of interest

The authors declare that the research was conducted in the absence of any commercial or financial relationships that could be construed as a potential conflict of interest.

## Publisher’s note

All claims expressed in this article are solely those of the authors and do not necessarily represent those of their affiliated organizations, or those of the publisher, the editors and the reviewers. Any product that may be evaluated in this article, or claim that may be made by its manufacturer, is not guaranteed or endorsed by the publisher.

## Supplementary material

The Supplementary material for this article can be found online at: https://www.frontiersin.org/articles/10.3389/fpubh.2023.1146531/full#supplementary-material

## References

[1] Tamir O Halkin H Shemer J. Generic drug substitution. Harefuah. (2006) 145:691–701. PMID: 17078434

[2] Shrank WH Choudhry NK Agnew-Blais J Federman AD Liberman JN Liu J. State generic substitution Laws can lower drug outlays under Medicaid. Health Aff. (2010) 29:1383–90. doi: 10.1377/hlthaff.2009.0424, PMID: 20606192 PMC3103121

[3] National Development and Reform Commission. (2015). Notice on the issuance of opinions on promoting drug Price reform (development and reform Price No. 904). Available at: https://www.ndrc.gov.cn/xxgk/zcfb/tz/201505/t20150505_963815.html?code=&state=123 (Accessed November 26, 2022)

[4] State Council. State council on the reform of the drug and medical device review and approval system. (2015). Available at: http://www.gov.cn/zhengce/content/2015-08/18/content_10101.htm (Accessed November 26, 2022)

[5] State Council. Opinions of the general Office of the State Council on the consistent evaluation of the quality and efficacy of generic drugs. (2016). Available at: http://www.gov.cn/zhengce/content/2016-03/05/content_5049364.htm (Accessed November 26, 2022)

[6] Huang B Barber SL Xu M Cheng S. Make up a missed lesson-new policy to ensure the interchangeability of generic drugs in China. Pharmacol Res Perspect. (2017) 5:e00318. doi: 10.1002/prp2.318, PMID: 28603636 PMC5464346

[7] IMS. Patents | IMS. (2020). Available at: https://imstsvc.com/patents/ (Accessed November 26, 2022)

[8] Sun J. International experiences of promoting generics use and its implications to China: promoting generics use. J Evid-Based Med. (2013) 6:74–80. doi: 10.1111/jebm.12030, PMID: 23829799

[9] Chen FC. China: the next pharmacy of the world? Trends Pharmacol Sci. (2018) 39:843–8. doi: 10.1016/j.tips.2018.07.004 30098798

[10] Lu S Cai J Xu F. Ultra-low price of generic agents in China may weaken patients’ drug recognition and compliance. Drug Discov Ther. (2020) 14:103–4. doi: 10.5582/ddt.2020.01011, PMID: 32295974

[11] Li W-J Xia M-J Gong S-W Ding Y-F. Perceptions of generic drugs in the pharmacists of public hospitals: a cross-sectional survey in Hubei Province of China. Curr Med Sci. (2021) 41:987–95. doi: 10.1007/s11596-021-2412-4, PMID: 34476663

[12] Aalto-Setälä V. The impact of generic substitution on price competition in Finland. Eur J Health Econ. (2008) 9:185–91. doi: 10.1007/s10198-007-0059-0, PMID: 17508226

[13] NMPA. (2021). Annual drug review report. Available at: https://www.nmpa.gov.cn/xxgk/fgwj/gzwj/gzwjyp/20220601110541120.html (Accessed November 26, 2022)

[14] Chen S Chen X Liu M Xu Z Yang Y. Regulation of generic drugs in China: Progress and effect of the reform of the review and approval system. J Pharm. (2022) 1–9. doi: 10.1007/s12247-022-09655-9

[15] Gupta R Shah ND Ross JS. Generic drugs in the United States: policies to address pricing and competition. Clin Pharmacol Ther. (2019) 105:329–37. doi: 10.1002/cpt.1314, PMID: 30471089 PMC6355356

[16] Frank RG Salkever DS. Generic entry and the pricing of pharmaceuticals. J Econ Manag Strategy. (1997) 6:75–90. doi: 10.1162/105864097567039

[17] Berndt ER Conti RM Murphy SJ. The landscape of US generic prescription drug markets, 2004–2016. (2017). Available at: https://www.nber.org/papers/w23640 (Accessed November 26, 2022)

[18] Nguyen NX Sheingold SH Tarazi W Bosworth A. Effect of competition on generic drug prices. Appl Health Econ Health Policy. (2022) 20:243–53. doi: 10.1007/s40258-021-00705-w, PMID: 34904207

[19] Dave CV Hartzema A Kesselheim AS. Prices of generic drugs associated with numbers of manufacturers. N Engl J Med. (2017) 377:2597–8. doi: 10.1056/NEJMc1711899, PMID: 29281576

[20] Reiffen D Ward MR. Generic drug industry dynamics. Rev Econ Stat. (2005) 87:37–49. doi: 10.1162/0034653053327694

[21] Zhang W Sun H Guh D Anis AH. The impact of price-cap regulations on market entry by generic pharmaceutical firms. Expert Rev Pharmacoecon Outcomes Res. (2017) 17:231–8. doi: 10.1080/14737167.2017.1271717, PMID: 27936981

[22] Wu J Xu J Liu G Wu J. Pharmaceutical pricing: an empirical study of market competition in Chinese hospitals. PharmacoEconomics. (2014) 32:293–303. doi: 10.1007/s40273-013-0099-5, PMID: 24190661

[23] Kobayashi E Karigome H Sakurada T Satoh N Ueda S. Patients’ attitudes towards generic drug substitution in Japan. Health Policy. (2011) 99:60–5. doi: 10.1016/j.healthpol.2010.07.006, PMID: 20685003

[24] Astier A. Interchangeability and substitution of biosimilars. Ann Pharm Fr. (2020) 78:277–84. doi: 10.1016/j.pharma.2020.04.004, PMID: 32387176

[25] China Government. Notice on the comprehensive reform of public hospitals in full swing. (2017). Available at: http://www.nhc.gov.cn/tigs/s3581/201704/c25c1e8eea794d71bece3bdbe6b8f8df.shtml (Accessed November 29, 2022)

[26] National Bureau of Statistics. National data. (2022). Available at: https://data.stats.gov.cn/search.htm?s=CPI (Accessed November 29, 2022)

[27] Olson LM Wendling BW. Estimating the causal effect of entry on generic drug prices using hatch-Waxman exclusivity. Rev Ind Organ. (2018) 53:139–72. doi: 10.1007/s11151-018-9627-y

[28] Tang M Song P He J. Progress on drug pricing negotiations in China. Biosci Trends. (2019) 13:464–8. doi: 10.5582/bst.2019.01339, PMID: 31875587

[29] Yuan J Lu ZK Xiong X Jiang B. Lowering drug prices and enhancing pharmaceutical affordability: an analysis of the national volume-based procurement (NVBP) effect in China. BMJ Glob Health. (2021) 6:e005519. doi: 10.1136/bmjgh-2021-005519, PMID: 34518200 PMC8438819

[30] Long H Yang Y Geng X Mao Z Mao Z. Changing characteristics of pharmaceutical prices in China under centralized procurement policy: a multi-intervention interrupted time series. Front Pharmacol. (2022) 13:944540. doi: 10.3389/fphar.2022.944540, PMID: 35910351 PMC9335887

[31] Zhang Y Wushouer H Han S Fu M Guan X Shi L. The impacts of government reimbursement negotiation on targeted anticancer medication price, volume and spending in China. BMJ Glob Health. (2021) 6:e006196. doi: 10.1136/bmjgh-2021-006196 PMC8286756 34266848

[32] Zhang H Zaric GS Huang T. Optimal Design of a Pharmaceutical Price-Volume Agreement under Asymmetric Information about Expected Market Size. Prod Oper Manag. (2011) 20:334–46. doi: 10.1111/j.1937-5956.2011.01219.x

[33] Xiaoshan H. [world saying] do drugs cost $130,000 a year? U.S. media: the U.S. drug prices are outrageously expensive the main responsibility lies with the government. China Daily. (2023). Available at: https://cn.chinadaily.com.cn/a/202302/27/WS63fc1784a3102ada8b230ca7.html (Accessed April 10, 2023)

[34] Zhao M Nie P Wu J. Heterogeneity in Price elasticity of medicine demand in China: moderate effect from economic incentive and quality difference. Front Pharmacol. (2021) 12:688069. doi: 10.3389/fphar.2021.688069, PMID: 34408651 PMC8366225

[35] Frank RG Mcguire TG Nason I. The evolution of supply and demand in markets for generic drugs. Milbank Q. (2021) 99:828–52. doi: 10.1111/1468-0009.12517, PMID: 34075623 PMC8452364

